# Clinical manifestations of lead-dependent infective endocarditis: analysis of 414 cases

**DOI:** 10.1007/s10096-014-2117-8

**Published:** 2014-05-03

**Authors:** A. Polewczyk, M. Janion, R. Podlaski, A. Kutarski

**Affiliations:** 1 II Department of Cardiology, Swietokrzyskie Cardiology Center, Kielce, Poland; 2 Department of Health Sciences, The Jan Kochanowski University, Kielce, Poland; 3 Department of Biology, The Jan Kochanowski University, Kielce, Poland; 4 Department of Cardiology, Medical University of Lublin, Lublin, Poland

## Abstract

It is important to identify clinical manifestations of lead-dependent infective endocarditis (LDIE), as it begins insidiously with the slow development of nonspecific symptoms. Clinical data from 414 patients with the diagnosis of LDIE according to Modified Duke Lead Criteria (MDLC) were analyzed. Patients with LDIE had been identified in a population of 1,426 subjects submitted to transvenous lead extraction (TLE) in the Reference Clinical Cardiology Center in Lublin between 2006 and 2013. The symptoms of LDIE and pocket infection were detected in 62.1 % of patients. The mean duration of LDIE symptoms prior to referral for TLE was 6.7 months. Fever and shivers were found in 55.3 % of patients, and pulmonary infections in 24.9 %. Vegetations were detected in 67.6 % of patients, and positive cultures of blood, lead, and pocket in 34.5, 46.4, and 30.0 %, respectively. The most common pathogens in all type cultures were coagulase-negative staphylococci (CNS), with *Staphylococcus epidermidis* domination; the second most common organism was *Staphylococcus aureus*. 76.3 % of patients were treated with empirical antibiotic therapy before hospitalization due to TLE. In the laboratory tests, the mean white blood cell count was 9,671 ± 5,212/μl, mean erythrocyte sedimentation rate 43 mm, C-reactive protein (CRP) 46.3 mg/dl ± 61, and procalcitonin 1.57 ± 4.4 ng/ml. The multivariate analysis showed that the probability of LDIE increased with increasing CRP. The diagnosis of LDIE based on MDLC may be challenging because of a relatively low sensitivity of major criteria, which is associated with early antibiotic therapy and low usefulness of minor criteria. The important clinical symptoms of LDIE include fever with shivering and recurrent pulmonary infections. The most specific pathogens were *Staphylococcus epidermidis* and *Staphylococcus aureus*. Laboratory tests most frequently revealed normal white blood cell count, relatively rarely elevated procalcitonin level, and significantly increased erythrocyte sedimentation rate (ESR) and CRP. This constellation of signs should prompt a more thorough search for LDIE.

## Introduction

Lead-dependent infective endocarditis (LDIE) is a serious and insidious disease developing in patients with cardiovascular implantable electronic devices [pacemaker (PM), implantable cardioverter defibrillator (ICD), cardiac resynchronization therapy (CRT)]. It is difficult to estimate the incidence of LDIE due to the vagueness of terminology and diagnostic challenges. Such terms as pacemaker endocarditis, cardiac device-related endocarditis, device-related infective endocarditis, and difficulties with detecting the disease prompt investigators to assess infectious complications as a whole (cardiac device infections, CDI), without separating out LDIE [1–5]. For about 20 years (1979–1999), the infection rate in patients with implanted permanent pacemakers was found to be 0.4–16.3 %, whereas LDIE was detected in less than 10 % of cases [1, 6–8]. In more recent reports, data are provided for different patient populations. Some of them estimate the presence of LDIE in patients with isolated infective endocarditis. An analysis of 2,760 patients with infective endocarditis in 61 centers from 28 countries (between 2000 and 2006) demonstrated LDIE in 177 (6.4 %) patients [9]. Other reports show significant differences in the rate of LDIE among infectious complications developing after PM/ICD implantation (ranging from 22 to 57 %) [2, 10–12]. The reason for this discrepancy is the above-mentioned vagueness of terminology and criteria for diagnosing LDIE. The nonspecific course of the disease, mimicking chronic lower respiratory disease and frequently not bringing to mind the implantable intracardiac device, adds to diagnostic difficulties. It concerns mainly isolated LDIE, as the coexistent pocket infection prompts extended diagnostics in search for LDIE. Moreover, available reports on LDIE comprised small numbers of patients, from 10 to 88 in single-center studies, and from 145 to 177 in multicenter registries [9, 10, 13–20]. For this reason, the clinical manifestations of LDIE remain vague and necessitate further research.

### Aim of the study

The aim of the present study was to analyze the clinical symptoms of LDIE in a large population of 414 patients undergoing transvenous lead extraction (TLE) because of LDIE.

## Methods

Clinical data from 414 patients with the diagnosis of LDIE were analyzed retrospectively and prospectively. Patients with LDIE had been identified in a population of 1,426 subjects submitted to TLE due to infectious and non-infectious complications in the Reference Clinical Cardiology Center in Lublin between March 2006 and July 2013. The patients were qualified for the study on the basis of Modified Duke Lead Criteria (MDLC) for the diagnosis of infective endocarditis on pacemaker leads taking into account two additional major clinical criteria of LDIE: pocket infection and pulmonary embolism, as well as pathological criteria regarding the presence of vegetations. According to the European Society of Cardiology (ESC) guidelines, definite infective endocarditis was diagnosed if two major or one major plus two minor or five minor criteria were met. Patients with probable LDIE, i.e., meeting one major plus one minor or three minor criteria, were also included in the analysis. The 414 patients with LDIE were analyzed for the following factors: primary indication for TLE, duration of symptoms until diagnosis, most frequent clinical symptoms (fever, shivers, pulmonary infections), antibiotic therapy, transthoracic echocardiography (TTE) showing the most frequent location of vegetations, and microbiological and inflammatory tests. Additionally, multivariate analysis of clinical symptoms and laboratory findings was performed to estimate the probability of LDIE.

### Statistical analysis

Continuous variables were expressed as means ± standard deviation. Student’s *t*-test was used to test for the significance of differences between the means. Qualitative variables were compared with a Chi-square test. The two-sided *p*-value ≤ 0.05 was considered significant. The statistics were calculated using STATISTICA version 10. Multivariate analysis was carried out to study the relationships between an explained (dependent) variable and many explanatory (independent) variables. Explanatory variables were identified using the Wald test, whereas collinearity was checked using the variance inflation factor (VIF) (McCullagh and Nelder 1989).

## Results

Most patients with the final diagnosis of LDIE had been initially diagnosed with LDIE (52.9 %); however, in 40.1 % of them, the primary reason for TLE was only pocket infection. Severe sepsis was detected in 1.2 % of patients, whereas 5.8 % of patients were referred for TLE without infectious complications.

**Fig. 1:**
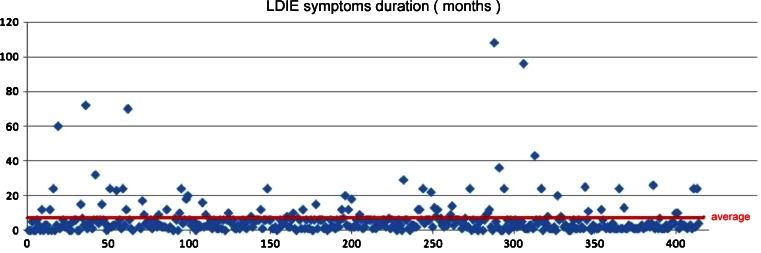
Time from the onset of symptoms to the initial diagnosis of lead-dependent infective endocarditis (LDIE)

The mean duration of LDIE symptoms before referral to transvenous lead removal was 6.7 months (±10.9), with the longest undetected LDIE being 108 months. In 18.8 % of patients, the diagnosis was made 6 months later and in 6 % at 20 months after the onset of the disease (Fig. 1).

In 62.1 % of patients, LDIE was concomitantly present with pocket infection.

**Fig. 2:**
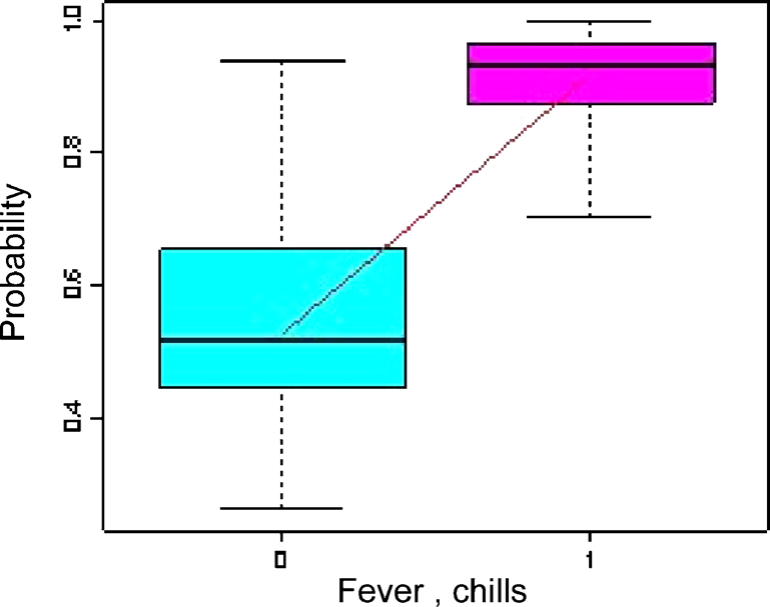
Multivariate analysis—fever and shivers as significant clinical features of LDIE

Fever and shivering were present in 55.3 %, whereas pulmonary infections were present in 24.9 % of patients. Multivariate analysis confirmed the significantly higher probability of diagnosing LDIE in patients with fever and shivering (a 5.8-fold increase) (Fig. 2).

**Fig. 3:**
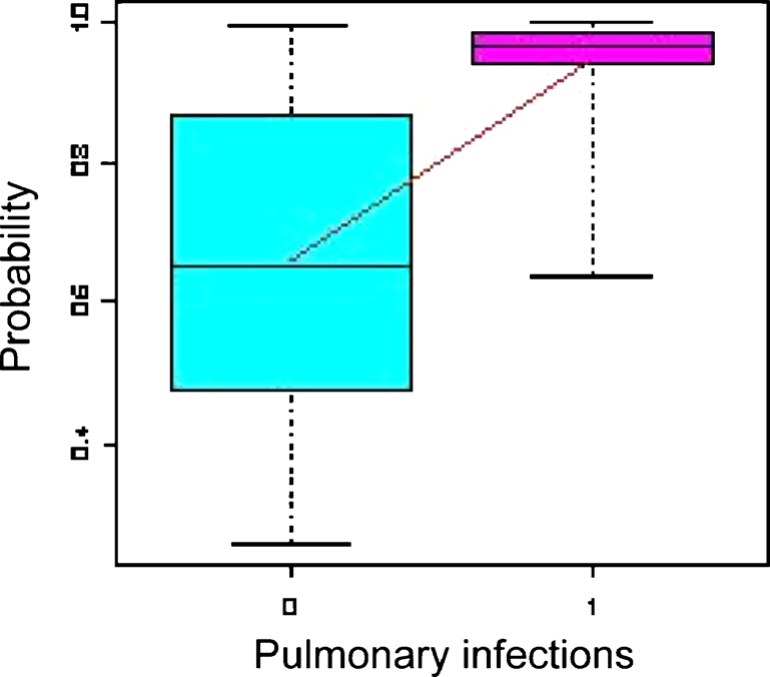
Multivariate analysis—increasing probability of pulmonary infections in patients with LDIE

Multivariate analysis showed also a 3.3-fold higher probability of pulmonary infections in patients with compared to patients without LDIE (Fig. 3).

**Fig. 4:**
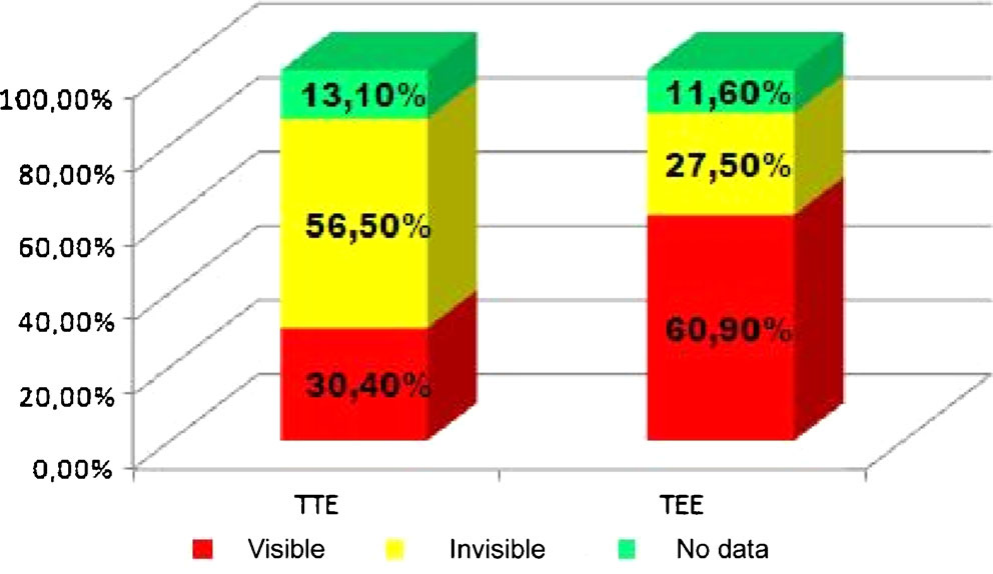
Transthoracic and transesophageal echocardiography (TTE and TEE, respectively) of vegetations in patients referred for transvenous lead extraction (TLE) between March 2006 and July 2013

Transthoracic and transesophageal echocardiography was performed in most patients with LDIE (TTE and TEE in 91 % of patients). Vegetations were confirmed in 67.6 % of patients with LDIE, including 30.4 % that were only detected with TTE. TEE revealed vegetations in 60.9 % of patients (Fig. 4).

Vegetations were most frequently located in the right atrium (81.8 %), including 27 % in the superior vena caval orifice. Vegetations were also seen in the right ventricle (14.7 %), near the tricuspid valve (9.1 %), and in the superior vena cava (7.9 %). In 15 patients (5.4 %), vegetations were concomitantly present in the left heart (Table 1).

**Table 1:** Location of vegetations in patients with lead-dependent infective endocarditis (LDIE) referred for transvenous lead extraction (TLE) between March 2006 and July 2013

Location of vegetations in patients with LDIE	%
Right atrium (RA)	81.8
Including vena cava superior (VCS) orifice	27
Right ventricle	14.7
Tricuspid valve	9.1
VCS	7.9
Concomitant left heart involvement	5.4

The concomitant presence of inflammatory process in the left heart was confirmed in 5.4 % of patients.

**Fig. 5:**
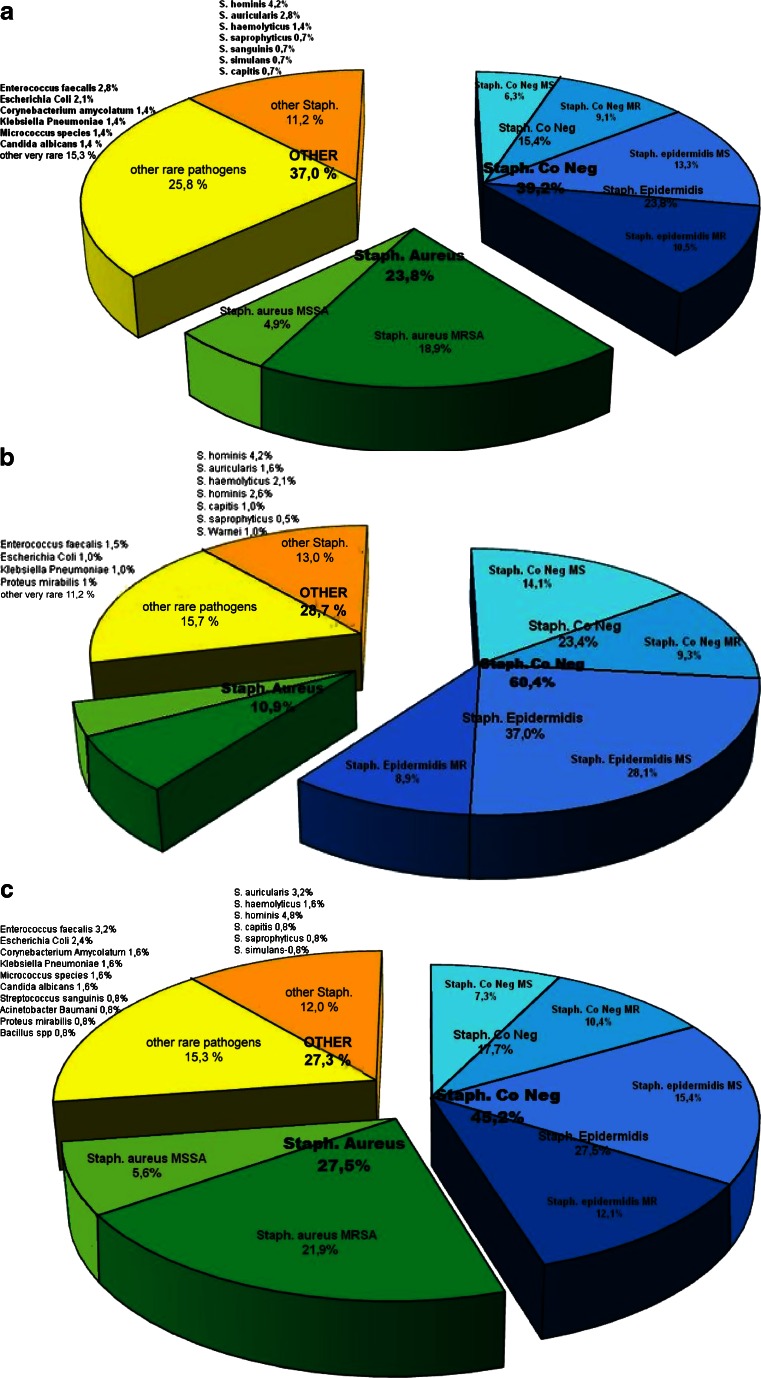
**a** Distribution of pathogens in blood cultures in patients with LDIE. **b** Distribution of pathogens in explanted lead cultures in patients with LDIE. **c** Distribution of pathogens in cultures of pacemaker pocket in patients with LDIE

Positive blood cultures were obtained from 34.5 % of patients with LDIE. The most common isolates were coagulase-negative staphylococci (CNS) [39.2 %, including methicillin-resistant coagulase-negative staphylococci (MRCNS) in 15.4 %]. In the CNS group, *Staphylococcus epidermidis* was the most common pathogen (23.8 %). *Staphylococcus aureus* was detected in 23.8 % of patients [including methicillin-resistant *Staphylococcus aureus* (MRSA) in 18.9 %]. *S. auricularis*, *S. capitis*, *S. haemolyticus*, *S. hominis*, *S. saprophyticus*, and *S. simulans* were decidedly less common (11.2 %), and there were only single cases of *Escherichia coli*, *Enterococcus faecalis*, *Candida albicans*, *Klebsiella pneumoniae*, and Corynebacteriacae (Fig. 5a).

Cultures of lead segments were positive in 46.4 % of patients. The most common isolates were CNS detected in 60.4 % of patients (including MRCNS in 9.3 % and *S. epidermidis* in 37 %) and *S. aureus* (10.9 %; MRSA in 8.9 %). *S. auricularis*, *S capitis*, *S. hominis*, *S. haemolyticus*, *S. saprophyticus*, and *S. warneri*, as well as *E. faecalis*, *K. pneumoniae*, *E. coli*, and *Proteus mirabilis*, were less common, with only single cases of other pathogens (Fig. 5b).

Cultures of generator pockets (positive in 30 %) in patients with LDIE contained CNS (45.2 %, including MRCNS in 10.4 %; *S. epidermidis* in 27.4 %) and *S. aureus* in 27.5 % of patients (MRSA in 21.9 %). *S. auricularis*, *S. capitis*, *S. hominis*, *S. haemolyticus*, *S. saprophyticus*, *S. simulans* and *E. faecalis*, *E. coli*, and *K. pneumoniae* were less common, with only single cases of other pathogens (Fig. 5c).

Antibiotic therapy before hospitalization because of TLE was used in 76.3 % of patients. The first-line treatment was most often broad-spectrum antibiotics; after positive culture, the therapy was modified and continued during TLE and after the procedure.

Of inflammatory markers measured in patients with LDIE, the white blood cell count was not found to be significantly elevated (mean 9,671 ± 5,212/μl). The mean procalcitonin levels were higher in the whole study population (1.57 ± 4.4 ng/ml). However, in 79.8 % of patients, the procalcitonin levels were low (<0.5 ng/ml). C-reactive protein (CRP) levels were significantly elevated in 75 % of patients (mean 46.3 mg/dl ± 61.2), similar to the erythrocyte sedimentation rate (ESR), which was high in 60.3 % of patients (mean 43 mm in 1 h ± 29.6) (Table 2).

**Table 2:** Inflammatory markers in patients with LDIE referred for TLE between March 2006 and July 2013

Laboratory tests	Result
White blood cell count (WBC) (mean ± SD)	9,671 ± 5,212
WBC >8,000/μl (%)	46.6
WBC <8,000/μl (%)	43.4
ESR, mm in 1 h (mean ± SD)	43 ± 29.6
OB >30 mm in 1 h (%)	60.3
OB <30 mm in 1 h (%)	39.7
CRP, mg/dl (mean ± SD)	46.3 ± 61.2
CRP >5.0 g/dl (%)	75
CRP <5.0 g/dl (%)	25
Procalcitonin (PRC) ng/ml (mean ± SD)	1.57 ± 4.4
PRC >0.5 ng/ml (%)	20.2
PRC <0.5 ng/ml (%)	79.8

**Fig. 6:**
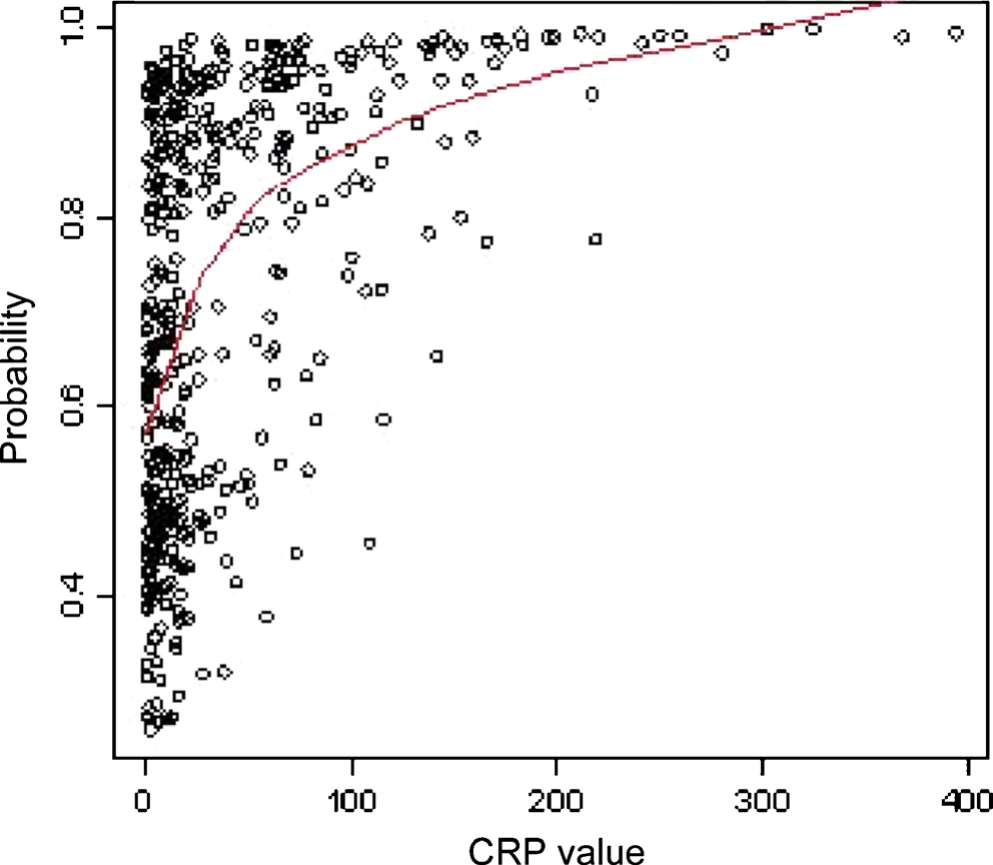
Multivariate analysis—increasing probability of elevated CRP in patients with LDIE

The multivariate analysis showed a significant relationship between elevated CRP levels and possible LDIE. The risk of LDIE increased by 0.8 % for a one-unit increase in CRP (Fig. 6).

## Discussion

The ESC guidelines on the prevention, diagnosis, and treatment of infective endocarditis recommend MDLC for the diagnosis of LDIE, taking into account positive blood culture, vegetations detected by echocardiography (associated with the presence of the leads), local infection, and pulmonary embolism confirmed by lung computed tomography (CT) or lung scintigraphy [21]. Nevertheless, the final classification of patients with LDIE is very difficult due to misleading symptoms and a variable and unpredictable course of the disease. LDIE is located in the right heart, and for this reason, respiratory symptoms dominate the clinical picture, i.e., cough, pleuritic chest pain, and recurrent pneumonia with atypical radiological signs. In this context, the presence of typical minor Duke criteria, characteristic of infected vegetation spread to the greater circulation, is rarely seen [22]. Observational studies in small populations (a total of 155 patients) confirm differences in the prevalence of symptoms: fever 51 to 80 % of patients, bacteremia 68 to 92 % of patients, concomitant pocket infection 22 to 80 % of patients [13, 23, 24]. Symptoms with relatively high specificity were shown in a multicenter study in 177 patients with LDIE in whom fever >38 °C occurred in 80.7 %, positive blood culture in 84.2 %, and vegetations in 89.8 % [9]. In the present study, fever and shivering were found in 55.3 % of patients, and multivariate analysis confirmed their significant role in the diagnosis of LDIE. The mean duration of LDIE symptoms from diagnosis to referral for TLE was 6.7 months, with the longest undetected LDIE being 108 months (9 years). Taking into account major Duke criteria of LDIE, vegetations were detected in 67.6 % of patients, positive blood cultures in 34.5 % (positive culture of extracted leads in 46.4 %), concomitant pocket infection in 62.1 % (with positive culture in 30 %), and recurrent lung infections (infected pulmonary embolism index) in 24.9 %. These results are similar to those obtained in small single-center studies [13, 23, 24]. Only one multicenter study demonstrated a significantly higher (>80 %) rate of fever, vegetations, and positive blood cultures [9]. In the present study, the sensitivity of TTE was low; vegetations were visualized in 30.4 % of patients. Observational studies show a similar rate of structural intracardiac abnormalities as one of the major criteria for the diagnosis of LDIE [14]. Difficulties in visualizing vegetations in TTE are caused mainly by their location. Although the present study showed the highest rate of vegetations in the right atrium (81.8 %), they were mainly located in the right atrium auricle and near the orifice of the superior vena cava (27 %), the areas that are not visible in TTE. Vegetations in the tricuspid area are also difficult to visualize because of lead-dependent reverberation. For this reason, it is extremely important to perform TEE. TEE in the present study detected vegetations in 60.0 % of patients. A recent observational study in 136 patients undergoing TLE for infective reasons demonstrated a similar location and frequency of vegetations in TEE [25].

Positive blood culture is another major Duke criterion of LDIE. As mentioned above, in the present study, positive blood cultures were found in 34.5 % of patients, which, in the light of antibiotic therapy used in 76.3 % of patients, should not be surprising. Due to the different masks of this disease (recurrent pneumonia, G+ sepsis unknown origin up to tuberculosis), most of the patients were previously treated with very different antibiotics in turn but with transient effect only. Unfortunately, long-standing antibiotic therapy often delayed proper diagnosis and TLE procedure. In microbiological studies, CNS dominated by *Staphylococcus aureus*. The results from previous studies vary, showing the prevalence of either CNS or *S. aureus*, both being the most common pathogens in patients with LDIE [11, 13–15, 23, 24, 26–29]. *S. auricularis*, *S. capitis*, *S. haemolyticus*, *S. hominis*, *S. sanguinis*, *S. simulans*, and *S. saprophyticus* (0.7–4.2 %), as well as *Candida albicans*, *Corynebacterium amycolatum*, *E. faecalis*, *E. coli*, *K. pneumoniae*, and *Micrococcus* species were less common, with only single cases of very rare pathogens. The statistics for these pathogens were also similar [11, 13–15, 23, 26, 28–30]. The prevalence of staphylococci, especially CNS, can be explained from the viewpoint of pathogenesis. These bacteria have specific properties that contribute to colonization of the leads. Adhesion is mediated by a bacterial surface component called MSCRAMM (microbial surface components recognizing adhesive matrix molecules) [31]. After initial adhesion to each other and to the host matrix, the bacteria multiply, forming a layer on the lead surface that is covered by an extracellular dense substance known as the biofilm matrix. Bacteria in biofilms resist antibiotics and host defenses [5, 32]. As LDIE is a specific variation of infective endocarditis associated with the presence of leads colonized by the pathogens, it appears justified to modify Duke criteria and take into account positive lead cultures in the diagnosis of the complication. However, there is still controversy surrounding LDIE. The current ESC guidelines on the prevention, diagnosis, and treatment of infective endocarditis propose that positive lead cultures can be used as a sign of LDIE only when the leads were removed in the absence of pocket infection [21]. It seems, however, logical to take into account all clinical features and gross examination of transvenously removed leads (purulent discharge from intracardiac leads and swabbing the proximal tips of the leads are not infrequent). As the material for culturing bacteria in the present study was obtained in a precise way, positive lead cultures were considered as an important major criterion of LDIE. Recent analysis of 417 infections confirms this approach [29].

Local pocket infection is still another major Duke criterion of LDIE. In the present study, LDIE coexisted with local pocket infection in 62.1 % of patients. In single studies in which LDIE was considered as an independent disease entity, pocket infection occurred in 46–70 % of cases [29, 30]. In the present study, all patients referred with local pocket infection were thoroughly diagnosed for the presence of LDIE, which increased the detection rate of the complication.

Pulmonary embolism is an additional major Duke criterion of LDIE. In the present study, the symptoms of recurrent lung infections (considered as markers of infected pulmonary embolism) were found in 24.9 % of patients with the final diagnosis of LDIE. In the multivariate analysis, recurrent lung infections were significantly associated with the presence of LDIE. The few studies demonstrated pulmonary embolism in 33 % of patients with LDIE, whereas recurrent lung infections were highly specific for the presence of vegetations in the lesser circulation [11, 33]. It seems that pulmonary embolism confirmed by vascular CT should be taken into account more often in patients with suspected LDIE, especially because of the fact that minor Duke criteria for the diagnosis of LDIE may be unreliable [26]. It is worth remembering that, initially, Duke criteria were defined for left-sided infective endocarditis, which has completely different clinical manifestations, and vegetations affecting the left side of the heart were infrequent in the present study (5.4 %).

Inflammatory markers, most frequently measured in the clinical assessment of LDIE, provide interesting information. In the present study, there was no significant increase in the white blood cell count. However, the ESR was significantly elevated the CRP level was markedly elevated in 75 % of patients, whereas the procalcitonin levels were rarely elevated. The multivariate analysis demonstrated a significant relationship between elevated CRP and LDIE. In one of the few studies measuring inflammatory markers in LDIE, the ESR, CRP, and white blood cell count were elevated [25]. Another analysis of 44 patients with LDIE confirmed the significantly elevated ESR and white blood cell count in over 50 % of patients [13]. However, another study demonstrated low white blood cell count but elevated CRP and ESR in a small group of 34 patients with cardiac device infections [10]. Clearly, further studies on the spread of infection based on inflammatory markers are warranted in order to define their specificity and sensitivity in all types of cardiac device infections.

## Conclusions

1. Analysis of the clinical manifestations of lead-dependent infective endocarditis (LDIE) revealed a nonspecific course of the disease. Vagueness of terminology, failure to separate out LDIE from most frequent infectious complications, difficulties in evaluating epidemiological characteristics, and true clinical symptoms delay markedly the diagnosis of LDIE.
2. The diagnosis of LDIE based on Modified Duke Lead Criteria (MDLC) remains challenging because minor criteria have no specificity. Major criteria also show a relatively low sensitivity of symptoms, most often because of early prehospital antibiotic treatment. The significance of fever, especially pulmonary symptoms, was confirmed in the multivariate analysis, and transesophageal echocardiography (TEE) was found to be important in order to improve the non-invasive detection of vegetations.
3. Delay in the diagnosis of LDIE of over 6 months in the present study confirms diagnostic difficulties and the importance of the thorough assessment of all symptoms occurring in patients with cardiovascular implantable electronic devices.
4. Blood, leads, and pocket cultures confirmed most often the presence of typical pathogens responsible for LDIE development—coagulase-negative staphylococci (CNS) and *Staphylococcus aureus*—according their specific properties that contribute to colonization of the leads.
5. Analysis of typical inflammatory markers revealed unelevated white blood cell count and procalcitonin levels in most patients, but significantly elevated erythrocyte sedimentation rate (ESR) and C-reactive protein (CRP). Furthermore, CRP was the only marker in the multivariate analysis that was significantly associated with increased probability of LDIE. This characteristic constellation of laboratory findings should prompt a search for LDIE.
